# The Exact Value of the Electrolytic Method

**Published:** 1884-08

**Authors:** 


### The Exact Value of the Electrolytic Method.

In a paper read before the American Academy of Medicine on the above-named subject (*Med. Record*, October 13th), the author, Dr. A. D. Rockwell, formulated the following conclusions: 1. The success met with in the treatment of malignant tumors is generally but trivial. In epithelioma, however, when superficial and easily reached, success may be had. 2. The electrolysis of *intramural* fibroids often reduces the size somewhat, and gives great relief. 3. For erectile and small cystic tumors electrolysis is a specific. 4. Goitres, if small and soft, may be reduced in size, even by external applications. Even when hard, electrolysis may be beneficial, but the results are variable. 5. Hairs can be permanently removed. 6. In many cases of stricture relief or cure can be obtained by electrolysis, but experience is not sufficient to speak of its value positively.

